# Satellite cell therapy – from mice to men

**DOI:** 10.1186/2044-5040-3-2

**Published:** 2013-01-31

**Authors:** Akshay Bareja, Andrew N Billin

**Affiliations:** 1 GlaxoSmithKline, 5 Moore Drive, Research Triangle Park, Durham, NC, 27709, USA; 2 Center for the Study of Aging and Human Development, Box 3003, DUMC, Room 3502 Busse, Building, Blue Zone, Duke South, Durham, NC, 27710, USA

**Keywords:** Stem cell, Satellite cell, Pax7, Therapy, Muscular dystrophy

## Abstract

Satellite cells are rare mononuclear skeletal muscle-resident cells that are the chief contributors to regenerative myogenesis following muscle injury. Although first identified more than 50 years ago, it is only recently that the murine satellite cell has become molecularly defined with the ability to prospectively isolate these cells from their niche. Human satellite cells are considerably less well understood with relatively few studies having been performed on them. In this review, a critical evaluation of this literature is provided along with a discussion of the practical and methodological issues involved with research on human satellite cells. The therapeutic potential of these and other cells types is also discussed, and the various challenges that face satellite cell therapy are addressed.

## Introduction

When Alex Mauro coined the term ‘satellite cell’ more than 50 years ago, his tentative and humble suggestions that these cells ‘indeed might be of interest to students of muscle histology’ and ‘might be pertinent to the vexing problem of skeletal muscle regeneration’ [1] belied not only their immense interest to a wide range of biologists but also their preeminent status as the key contributors to adult muscle regeneration. Satellite cells are mononuclear cells with low cytoplasmic content that are located between the basal lamina and sarcolemma of adult skeletal muscle fibers [1]. Under normal conditions, these cells are mitotically quiescent and in response to injury become activated, proliferate and differentiate into myocytes that eventually fuse with each other or with existing myofibers to generate new muscle tissue [2]. At least a portion of the satellite cell pool is able to self-renew and is, therefore, considered a population of *bona fide* stem cells. The ability of these cells to self-renew and form new muscle tissue offers tremendous therapeutic opportunity in conditions of muscle disease or loss.

While our understanding of murine satellite cell biology is rapidly expanding, human satellite cells are considerably less well understood, with relatively few studies having been performed on them (see Table 1). The human satellite cell literature is also riddled with imprecise and, hence, confusing nomenclature. For the purposes of this review, the term ‘satellite cell(s)’ is taken to represent the total population of sublaminar/sarcolemma-adjacent cells. The prospective isolation techniques in general use are assumed to isolate a portion of this total population, although it is difficult to determine experimentally the precise fraction of total satellite cells isolated. Further, subsets of the population are divided into satellite stem cells (predominantly self-renewing) and satellite muscle progenitor cells (predominantly producing myoblasts) [3]. With respect to these definitions and considerable published literature, we suggest that satellite cells in culture begin to exit the quiescent state soon after seeding producing mostly myogenic progenitors and rarely dividing to self-renew. Therefore, such cultures are more appropriately termed satellite cell initiated cultures [4,5].

**Table 1:** Comparison of murine and human satellite cells

**Category**	**Mouse**	**Human**
Identifiable on muscle sections?	Yes By EM [6] By IHC; markers include Pax7 [21] and M-cadherin [22]	Yes By EM [7] By IHC; markers include Pax7 and CD56 [24]
Isolation of myofiber with associated satellite cells?	Yes [27]	Yes [26]
Identify live cells in culture?^a^	Yes [17,33-36]	Yes [61,62]
Intramuscular abundance	Less than 5% [6]	about 2% [9]; about 4% [7,8]
Gene signatures of satellite cells?^a^	Yes [63,64]	Yes [65]
Contribution to muscle growth following transplantation?	Yes [18,19,66]	?
Formation of functional satellite cells *in vivo* following transplantation?	Yes [18,19,66]	?
Satisfy criteria for being *bona fide* stem cells?	Yes [3,17]	?

^a^Although the authors of all the cited papers refer to their isolated cells as ‘satellite cells’, this designation is often not the same as the definition used here. Please refer to the main text for a detailed discussion. EM, electron microscopy; IHC, immunohistochemistry.

### Satellite cells and muscle regeneration

Satellite cells display a marked decrease in number from birth onwards. Electron microscopy revealed that in mice satellite cell nuclei account for 30% to 35% of total myofiber nuclei at birth but less than 5% in adult muscle [6]. Similar values have been obtained for human adult muscle (4%, 4.4%, and 2% [7-9]). Although actively contributing to muscle growth in juveniles [10], satellite cells are present in a quiescent state in adult muscle [11]. Satellite cells can be roused from this state of quiescence in response to exercise [12], muscle stretch [13,14] and injury [15]. Once activated, satellite cells follow a well-characterized proliferation and differentiation pathway, and have been shown to completely regenerate new myofibers within four days [16]. Although other cell types display varying degrees of myogenic potential (discussed later in this review), satellite cells are the only muscle-resident cell population to exhibit all of the properties of fully myogenic muscle stem and progenitor cells, including robust myofiber regeneration in injured muscle and engraftment of the satellite cell compartment following intramuscular injection [5,17-19].

### Identification and isolation of satellite cells

**Figure 1:**
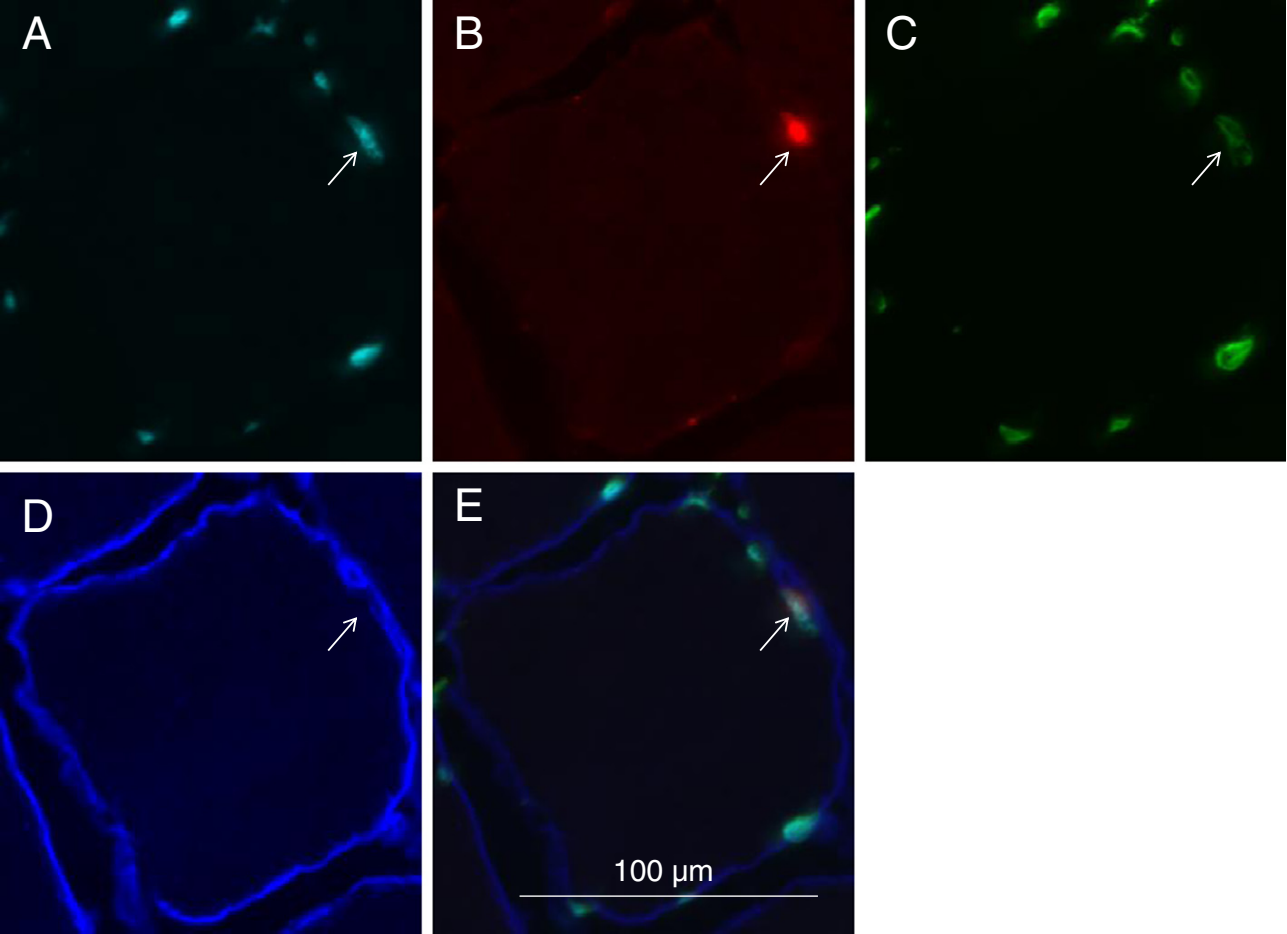
**Satellite cell in cross-section of human myofiber. A**: Hoechst 33342 staining indicating nucleus. **B**: Pax7 staining indicating satellite cell. **C**: Lamin A/C staining indicating nuclear membrane surrounding the satellite cell. **D**: Laminin staining indicating basal lamina surrounding the myofiber. **E**: Composite of images A to D.

The discovery and early identification of satellite cells was made possible by the use of electron microscopy [1], which is a method that is still used today to reveal ultrafine structural details [20]. The next breakthrough in our understanding of the satellite cell compartment came with the use of immunohistochemistry on muscle sections to visualize characteristic expression markers with fluorescence microscopy. These studies uncovered proteins enriched in the satellite cell associated with purified fibers such as Pax7 [21], CD34 [22], and specifically in humans, CD56 [23] among many other markers (reviewed extensively in [2]). It has become routine for these stains to be performed along with antibodies to laminin, which is a constituent of the basal lamina that surrounds each muscle fiber and allows for the correct identification of satellite cells as residing underneath the basal lamina [24] (Figure 1). Given that such tools and techniques are in general use, human satellite cells can be readily identified in tissue sections [8] and by flow cytometry of muscle derived fixed cell suspensions [25]. Intact, isolated single fiber staining has also been used to identify human satellite cells in much the same way as mouse satellite cells have been identified in this preparation. This technique has recently been used to identify satellite cells on human myofibers isolated from biopsies [26].

Despite the availability of numerous histological markers, the correct isolation of a pure population of human satellite cells has remained elusive. Skeletal muscle is a heterogeneous mix of cell populations which makes contamination of heterologous stem cells a difficult problem to circumvent. Additionally, studies claiming to have isolated human satellite cells have lacked sufficient experimental demonstration of the isolated cells’ properties. We believe that in order for a cell population to qualify as genuine satellite cells, the following *in vitro* and *in vivo* experimental tests must be satisfied. *In vitro*, they must be able to form myogenic colonies when cloned as single cells and form myogenic cultures when cultured *en masse*; 2) upon transplantation into the muscle environment they must contribute to new muscle formation *in vivo*; 3) upon transplantation they must occupy the satellite cell niche *in vivo* and expand over time in the niche; and 4) post-transplantation they must be able to undergo re-isolation from host muscle and still fulfill the above criteria.

Popular methods of isolating satellite cells include the removal and culturing of individual myofibers that harbor satellite cells, enzymatic or non-enzymatic liberation of satellite cells from muscle tissue and fluorescence-activated cell sorting (FACS) of satellite cells using specific cell-surface markers. Richard Bischoff developed the technique of isolating and culturing individual myofibers from rat flexor digitorum muscle. Scanning electron micrographs of these myofibers clearly revealed the presence of attached satellite cells [27]. This method has since been used on other skeletal muscles [28-30]. An obvious limitation of the use of this method to study human satellite cells is that it is only applicable to small muscles, as whole, undamaged myofibers need to be isolated and cultured.

Pre-plating and differential centrifugation are two methods of isolating satellite cells from muscle derived cell suspensions. Following the physical and enzymatic breakdown of muscle tissue, pre-plating is used to obtain an enriched population of satellite cells by removing fibroblasts that adhere more strongly to the plastic in tissue culture flasks than do satellite cells. The major disadvantage of both these techniques is that they do not guarantee an entirely pure population of satellite cells [31]. Also, as satellite cells typically become activated soon after isolation, thereby losing much of their therapeutic regenerative potential [32], use of this technique is inappropriate for isolating quiescent satellite cells as it typically takes many days to perform.

The most sophisticated method currently being used to isolate satellite cells is FACS. Knowledge of satellite cell-specific surface markers has been exploited by using fluorochrome-conjugated antibodies to discriminate and separate these cells from others following enzymatic and physical breakdown of muscle tissue. The most successful methods make use of antibodies that bind to both positive (proteins specific to satellite cells) and negative (proteins that are expressed by contaminant cells) markers. Different groups have used different combinations of markers, with comparable results from mouse muscle. For example, the marker sets CXCR4+, β1-integrin+, CD45-, Sca1-, Mac1-; α7-integrin+, CD34+, CD45-, CD31-, CD11b-, Sca1-; and α7-integrin+, CD45-, CD31-, Sca1- have all been used to isolate pure populations of cells that display the morphological and phenotypic properties of satellite cells and that regenerate damaged muscle tissue when injected into mouse muscle [17,33,34]. In addition, some investigators have used VCAM1 and Syndecan3/4 antibodies in combination with the negative selection markers listed above for the prospective isolation of murine satellite cells [35,36]. Antibodies against Pax7 have been used to isolate satellite cells from human muscle biopsies [25]. However, as Pax7 is an intracellular protein, it cannot be used to isolate live satellite cells.

Pisani *et al*. have demonstrated the power of this technique by showing that even a single marker (CD34) can be used to separate cells derived from human muscle biopsies with myogenic potential (CD34-) from cells with adipogenic potential (CD34+). In addition to deepening our understanding of the different cell types that reside in human skeletal muscle, this finding is of therapeutic value as it might be desirable to eliminate cell types with adipogenic potential when performing cell therapy for treating muscle diseases, such as Duchenne muscular dystrophy (DMD), which result in harmful fat accumulation [37]. However, it must be noted here that so-called fibro/adipogenic progenitors have been shown to have pro-myogenic effects on satellite cells in the mouse, and could, therefore, be of possible therapeutic value [38,39]. The FACS technique has been used by Pisani *et al*. to identify a rare population of multipotent adipomyogenic cells (CD34+CD15+CD56+) that can give rise to committed adipogenic (CD34+CD15+CD56-) and myogenic (CD34+CD15-CD56+) progenitors. These multipotent progenitors are located in the interstitial compartment and do not express *Pax7*, so are, therefore, distinct from satellite cells [40]. Nevertheless, these promising findings suggest that we are close to establishing a set of surface markers that can be used to prospectively isolate a pure population of human satellite cells. The identification of such a set of antibody markers will, however, not suffice to declare that the isolated cells are satellite cells. As in the mouse system, the isolated cells must have the functional properties of a satellite cell. Complicating these experiments will be the need to rely on immunocompromised mice as hosts for the cells and lack of genetic cell fate markers as have been used in mice.

The isolation of human satellite cells will allow for the first time a direct side-by-side comparison with mouse satellite cells. While it is generally assumed that mouse and human satellite cell biology is congruent, there is currently little direct experimental evidence supporting this assumption.

### Therapeutic opportunity of satellite cells

The chronic and debilitating loss of muscle function is a problem that afflicts many people. Loss of function can be the result of atrophy (which refers to the reduction of muscle mass of genotypically normal muscle, of which there can be many causes) or dystrophy (which in the context of muscle physiology refers to loss of functional muscle caused, usually, by a genetic defect). A third kind of muscle function loss may be the result of traumatic injury that damages large sections of muscle called volumetric muscle loss [41]. Two major types of atrophy are cachexia and sarcopenia. Cachexia is a multifactorial syndrome that involves the sudden loss of body fat and muscle as the result of disease (such as cancer or AIDS), while sarcopenia is defined as age-related muscle loss [42]. Of the many muscular dystrophies identified, DMD is one of the most severe and the most common. DMD is an X-linked recessive disease that afflicts 1 in every 3,300 boys and is caused by the absence of the protein dystrophin. It is a severely debilitating disease with many patients dying of respiratory failure in their twenties [43]. Muscle loss due to trauma is a much more diffuse group of syndromes but still very debilitating for those suffering from such injuries. In terms of therapeutic approaches invoking the activity of satellite cells the muscular dystrophies and traumatic injuries are more likely to benefit than atrophy conditions because the former are diseases or conditions in which regeneration of muscle tissue is compromised or insufficient. In fact, recent studies suggest that satellite cell activity may not be needed for certain kinds of muscle hypertrophy, further casting doubt on the potential efficacy of therapies for atrophy aimed at increasing the activity of satellite cells [44,45].

Of the many therapeutic strategies that are currently being tested to combat muscular dystrophy and to promote muscle regeneration, cell therapy is an approach that has received much attention and shows promise. This strategy involves the delivery of cells that make new muscle to diseased areas. These can either be muscle precursor cells or stem cells that have the ability to differentiate into muscle cells. Myoblast transfer (MT) is the oldest cell therapy approach and involves the derivation of myoblast cells from healthy donor skeletal muscle, expansion of these cells in culture, and administration to dystrophic tissue. Despite initially promising results, this approach has been plagued by many problems, namely poor migration of these cells and the need for immunosuppressants, which could have toxic effects and actually kill the myoblasts themselves [46]. There is, therefore, clearly a need to find an alternative type of cell, which has more potent myogenic effects. Ideally, this cell type would also have the ability to self-renew. Satellite cells are an ideal candidate as they not only have the ability to generate new muscle effectively but are also able to create new copies of themselves [17,32].

### Therapeutic challenges for satellite cell therapy

Establishing a reliable method of isolating a pure population of human satellite cells is only the first step towards developing a therapy for muscle disease. Among the many hurdles that need to be circumvented is the production of a sufficient number of cells for an effective, whole-body therapy. Satellite cells are rare, accounting for less than 5% of total myofiber nuclei in humans [8]. This problem is compounded by the fact that muscle biopsies tend to be very small. There is currently debate about the minimum number of cells needed for an effective therapy. There is evidence that significantly fewer satellite cells are needed to regenerate muscle tissue compared to myoblasts. Remarkably, transplantation of even one satellite cell is sufficient to give rise to new myofibers and satellite cells [5]. Despite this impressive finding, it is very likely that many more satellite cells will be required as multiple muscle groups will need to be targeted in patients. Unfortunately, *ex vivo* expansion of murine satellite cells significantly impairs *in vivo* engraftment potential following transplantation [32]. Therefore, a major challenge is to increase the number of satellite cells in culture while ensuring that they retain their potent regenerative and self-renewal properties. Further understanding of the satellite cell’s niche will aid in the maintenance of stem cell regenerative and self-renewal properties *in vitro*.

The term ‘niche’ refers to a restricted, complex milieu that impinges on stem cell survival and function. It refers to anatomical position and biophysical properties associated with that position, signaling molecules (such as mitogens, myokines and growth factors) and surrounding cells and tissue. A pivotal series of experiments was performed by Carlson and Faulkner, who showed that when limb muscles from old rats were transplanted into the limbs of young mice they displayed significant improvement in mass and force production compared to muscle transplanted into old hosts, thereby highlighting the importance of the muscle environment to muscle maintenance and function [47]. It has since been shown that aged satellite cells possess the latent ability to effectively proliferate and regenerate muscle, which can be reawakened after exposure to FGF (fibroblast growth factor) [29] and serum from young mice [48]. In addition to local biochemical influences, satellite cells have also been shown to be affected by the physical properties of the surrounding tissue. *Ex vivo* experiments on freshly-obtained satellite cells have revealed that their proliferative capacity is influenced by the elasticity of the substrate on which they are cultured, while their ability to differentiate is influenced by both elasticity and substrate protein composition [49]. Of note is a recent study that reveals the surprising finding that muscle stem cells are enriched in post-mortem tissue, thus revealing another potential source of satellite cells that could be of therapeutic use [50].

Another possible therapeutic challenge is immune rejection of transplanted allogeneic cells and/or resulting myofibers. This is a problem that has hampered many myoblast transfer trials and has necessitated the use of immunosuppressants. A recent study highlights dramatic levels of immune rejection following intramuscular injection of myoblasts into macaques. The authors suggest that sarcolemmal damage is caused by the infiltration of CD8+ lymphocytes [51]. However, Hall *et al*. have shown that the delivery of as few as three to five myofibers (with resident satellite cells attached) to immunocompetent mouse tissue results in long-term (up to 21 months) engraftment and tissue regeneration [18]. Although the authors do not provide an explanation for why the foreign tissue was not rejected, this is nonetheless a promising result that warrants further research.

Another potentially confounding issue is that of heterogeneity of satellite cells, which has been extensively reviewed elsewhere [52]. In mice, approximately 10% of satellite cells have never expressed the myogenic factor *Myf5*, and these have been termed ‘satellite stem cells’ because they have more potent engraftment potential and ability to self-renew than Myf5+ cells [3]. It is, therefore, imperative that similar studies be performed in humans to possibly identify a sub-population of satellite cells that are more therapeutically relevant.

### Therapeutic potential of other cell types

In mice, many other cell types, both within and without skeletal muscle, have been shown to display varying degrees of myogenic potential. Two of these cell types that have also been extensively studied in humans are mesoangioblasts and pericytes, which are both vessel-associated cells. Mesoangioblasts were first shown to be of therapeutic value when injected into alpha-sarcoglycan-null mice and partially correcting the dystrophic phenotype [53]. The major advantage these cells have over satellite cells is their ability to cross blood vessel walls, making systemic delivery far more feasible. Promising results from animal studies have led to Phase I clinical trials in DMD patients [54]. A recent study has revealed the possibility of genetically engineering mesoangioblasts to carry a human artificial chromosome (HAC) vector that carries the full-length dystrophin gene. Both intramuscular and intra-arterial injection of these modified cells into dystrophin-deficient *mdx* mice resulted in the formation of new, dystrophin-expressing myofibers. These cells were also shown to be able to contribute to the resident satellite cell population [55]. This study, therefore, encourages the isolation of autologous mesoangioblasts from DMD patients, followed by genetic correction and re-injection of these cells into the same patients. There is a well-established protocol for isolating mesonagioblasts from human muscle biopsies [56]. However, as this protocol is technically similar to the pre-plating technique described earlier, it is less likely to consistently produce a pure population of cells than a FACS-based prospective isolation technique. Pericytes isolated from human muscle have been shown to have *in vitro* myogenic potential and the ability to give rise to new dystrophin-positive myofibers when injected. These cells have been shown to be easily expandable in culture and amenable to genetic correction (in this case, transduction of a lentiviral vector carrying a mini-dystrophin gene) [57].

Additional sources of myogenic cells are embryonic stem (ES) and induced pluripotent stem (iPS) cells. The major advantage that these cells have over the other cell types mentioned in this review is that they can be extensively expanded in culture while maintaining their self-renewal and pluripotent properties. Induced expression of *PAX7* in both human-derived ES and iPS cells has been shown to produce myogenic precursors that, when transplanted via intramuscular injection into dystrophin-deficient mice, resulted in the sustained formation of human dystrophin-positive myofibers and even replenishment of the satellite cell compartment [58]. The iPS cells in this study were generated from resident fibroblasts. This strategy therefore has the advantage of generating an unlimited number of autologous myogenic cells that can be re-injected into the patient without concern about immune rejection. iPS cells offer the additional advantage of being amenable to genetic correction. A recent study has shown that fibroblast cells derived from patients with limb-girdle muscular dystrophy type 2D (LGMD2D) can be converted to iPS cells by overexpression of the reprogramming factors OCT3/4, KLF4, SOX2 and cMYC (the so-called ‘Yamanaka factors’). These cells were then used to generate mesoangioblast-like cells which were subsequently transduced by lentiviral vectors carrying the *SGCA* gene (which is defective in LGMD2D patients) and an inducible version of MyoD (to enhance myogenic differentiation). Intramuscular and intraarterial injection of these cells into immunocompromised *SGCA*-null mice resulted in a significant increase in SGCA+ myofibers and an improvement of the functional properties of the targeted muscle (tibialis anterior) [59]. However, the risk of teratoma formation does warrant caution when considering these cells for clinical trials.

## Conclusions

A recent study cemented the satellite cell’s status as the key contributor to muscle regeneration by showing that, in mice, Pax7+ cells were solely responsible for muscle mass recovery following acute injury [60]. Although these results do not rule out the potential therapeutic value of other cell types, they do provide further evidence that a satellite cell-based therapy is an area of investigation worth pursuing. Many pivotal studies on murine satellite cells have been conducted in the last decade that have paved the way for similar work that should be done on human satellite cells. Satellite cells could be tested alone or in combination with the other cell types mentioned in this review. The fact that numerous MT clinical trials have been performed means that many of the methodological problems involved with human cell therapy have already been addressed and suggests that new therapies may be expeditiously tested.

## Abbreviations

DMD: Duchenne muscular dystrophy; ES: Embryonic stem cell; FACS: Fluorescence activated cell sorting; FGF: Fibroblast growth factor; HAC: Human artificial chromosome; iPS: Induced pluripotent stem cell; LGMD2D: Limb-girdle muscular dystrophy type 2D; MT: Myoblast transfer.

## Competing interests

The authors declare they have no competing interests.

## Authors’ contributions

AB and ANB wrote the manuscript. Both authors read and approved the final manuscript.
